# Improved usability of an active-polarized 3D display in exoscopic surgery under non-optimal viewing conditions

**DOI:** 10.1007/s00405-026-10177-0

**Published:** 2026-04-23

**Authors:** Ryuhei Okada, Keiji Honda, Koharu Nakayama, Takeshi Tsutsumi, Takahiro Asakage

**Affiliations:** 1 Department of Head and Neck Surgery, Institute of Science Tokyo, Tokyo, Japan; 2 Department of Otorhinolaryngology, Institute of Science Tokyo, Tokyo, Japan; 3 Medical Solutions Business Div. Life Science & Technology Business Unit, Sony Corporation, Tokyo, Japan

**Keywords:** 3D display, Active polarizer, ORBEYE, VITOM, Surgery, Target-tracking

## Abstract

**Purpose:**

3D exoscopes have been increasingly adopted in otorhinolaryngology. However, conventional passive-polarized 3D displays (PPD) have limitations in vertical viewing angle, which can impair 3D visualization at nonfrontal angles, particularly for surgical assistants. Active-polarized 3D displays (APD) can overcome these limitations. This study aimed to compare the usability and performance of a conventional PPD and a prototype APD using the ORBEYE 4 K 3D exoscope system under viewing conditions unfavorable to the PPD.

**Methods:**

Twenty-four otorhinolaryngologists participated in the study. A prototype APD and a commercially available PPD were connected in parallel to the ORBEYE. The participants performed a procedural task simulating stapes surgery with the viewing position set 20° above the display center. The task performance was evaluated based on the number of successfully completed procedural tasks. A target-tracking test was performed before and after the procedural task to evaluate ocular fatigue by calculating the slope of the saccadic main sequence. The usability was assessed using a questionnaire.

**Results:**

The APD scored higher than the PPD for all questionnaire items. The number of successful procedural tasks was significantly higher in the APD group. With the APD, there was no change in perceived ocular fatigue before and after the procedural task, whereas with the PPD, there was a tendency toward increased fatigue.

**Conclusion:**

The APD demonstrated superior usability and task performance compared to a conventional PPD, particularly under vertically displaced viewing conditions. APD may be particularly beneficial for assistants and surgeons working at various levels of the eye.

**Supplementary Information:**

The online version contains supplementary material available at 10.1007/s00405-026-10177-0.

## Introduction

Since the 2010s, exoscopes such as the VITOM system (Karl Storz GmbH & Co.) have been used in neurosurgical procedures, which were previously dominated by conventional surgical microscopes [1–3]. Exoscopes have a longer focal length than endoscopes, and displaying the operative field on a display positioned in front of the surgeon enables the surgery to be performed in a more natural posture, which is their greatest advantage. Initially, exoscopes were limited to 2D visualization. However, in the late 2010s, 3D exoscopes were introduced, further enhancing their usefulness (VITOM-3D, Karl Storz GmbH & Co.) [4]. The image quality was subsequently improved to 4 K resolution, providing high-definition visualization. The 4 K 3D exoscope ORBEYE (Sony Olympus Medical Solutions) has been widely used in Japan since the late 2010s [5]. Exoscopes were initially introduced in neurosurgery; however, their use has gradually expanded into otorhinolaryngology. They were first adopted for temporal bone surgery [6, 7] and later extended to procedures involving the thyroid gland [8, 9], salivary glands [10, 11], and other areas that had not traditionally been examined with a surgical microscope. High-quality 3D imaging enables more delicate surgical procedures, and in addition, the ability for multiple individuals to share the same 3D image is considered to offer substantial educational advantages [3, 12].

To generate depth perception, 3D exoscopic surgery requires a stereoscopic display that delivers separate images to the left and right eyes. Most currently available surgical 3D displays employ a passive-polarization-based line-by-line method. In this method, left- and right-eye images are assigned to alternating horizontal pixel rows and separated by polarization filters in both the display and the viewer’s glasses. Consequently, each eye receives only half of the original vertical resolution, and accurate stereoscopic perception is maintained within a limited vertical viewing angle. This restricted vertical viewing zone becomes particularly problematic during standing procedures, such as thyroid and parotid gland surgery, in which the surgeon’s viewing height frequently changes. Under these conditions, surgeons may experience visual discomfort and fatigue, or may be forced to adopt constrained head positions to preserve stereoscopic vision. In addition, assistants and observers positioned away from the optimal viewing axis are more likely to experience degraded 3D perception and asthenopia [5].

**Fig. 1:**
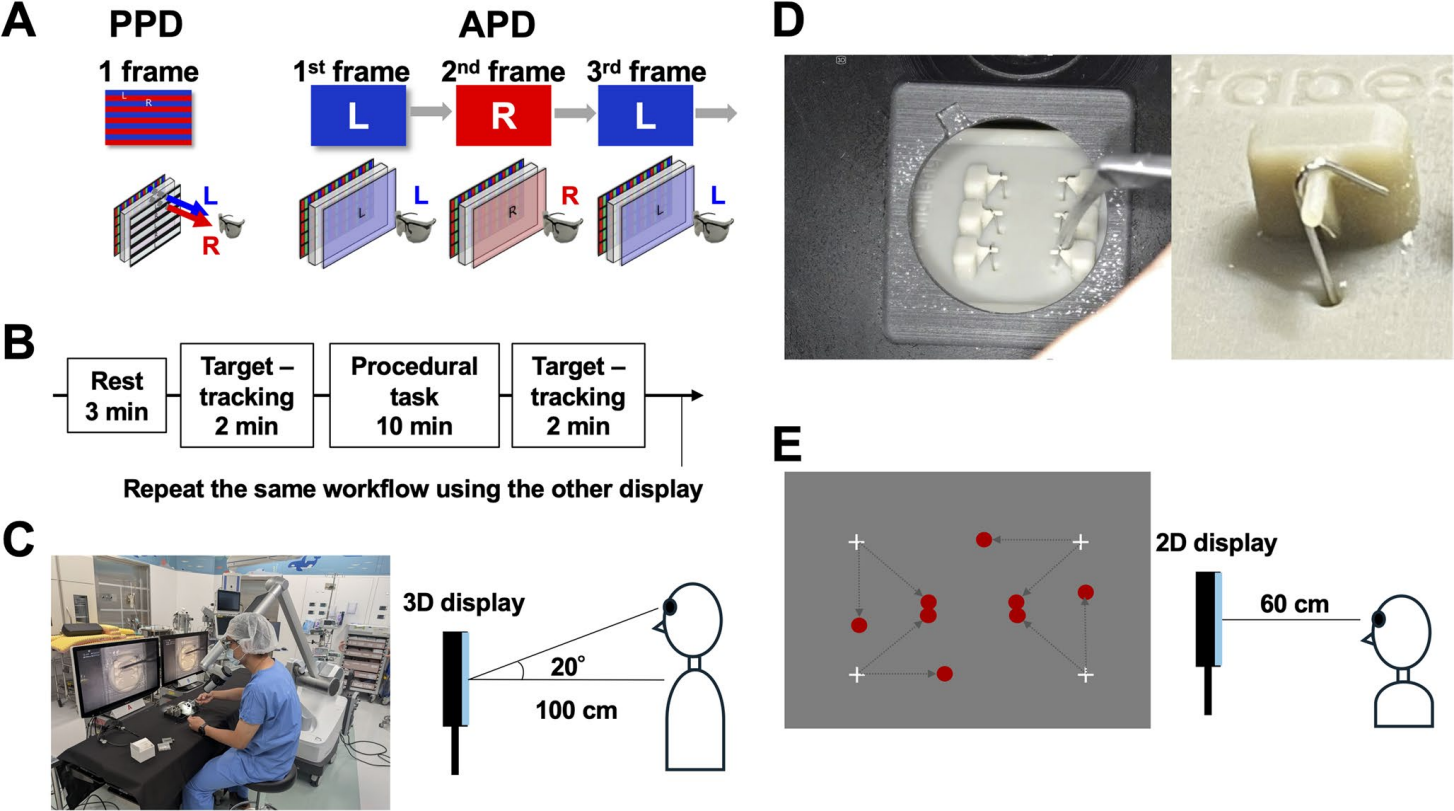
Experimental methods. (**A**) In a conventional passive-polarized 3D display (PPD), images for the left and right eyes are arranged in an alternating vertical pattern. In contrast, a prototype active-polarized 3D display (APD) projects images for the left and right eyes across the entire screen and switches between them instantaneously. (**B**) After receiving an explanation and practicing the procedural task for 5 min, participants took a 3-minute break, performed the target-tracking task for 2 min, completed the procedural task for 10 min, and then performed the target-tracking task again for 2 min. The displays were then switched, and the same sequence was repeated. The starting display (PPD or APD) was randomized so that half of the participants began with each display. (**C**) Viewing position during the procedural task. The participants’ viewing position was set 100 cm horizontally from the 3D display, at a point 20° above the center of the screen. (**D**) Procedural task. The Stapes Challenge of OtoSkills Trainer (Grace Medical, USA) was used. (**E**) The target-tracking task was performed to assess oculomotor fatigue. The participants’ viewing position was set 60 cm from a 2D display, at the same height as the display’s center. Participants were asked to track alternating white crosses and red dots, and their eye movements were recorded using an eye tracker during the task

An active-polarized 3D display (APD) presents alternating left- and right-eye images over the entire screen at high temporal frequencies (Fig. 1A). This approach eliminates the limitations of viewing angle and viewing distance inherent to conventional passive-polarized 3D displays (PPDs) and enables full-resolution image delivery to each eye, theoretically doubling the effective spatial resolution compared with PPDs. APD is therefore considered particularly useful when the viewing position deviates from the optimal frontal axis, where PPDs fail to provide stable 3D visualization, and to reduce the visual burden on assistants who cannot view the display directly from the front. This study was conducted to compare the usability of an APD and a PPD when the viewing position was above the display.

## Materials and methods

Details are provided in sData 1.

### Display settings, participants, and experimental procedures

A 32-inch APD prototype was developed, and a commercially available 32-inch PPD (LMD-XH320MT, Sony) served as the control. Both displays were connected in parallel to the ORBEYE 4 K 3D exoscope to provide identical video output.

Twenty-four otorhinolaryngologists and head and neck surgeons participated in this study (median age: 37 years; range: 26–59). This was an exploratory study and no formal sample-size calculation was performed. After 5 min of practice without the exoscope, the participants completed a procedural as well as target-tracking tasks in a cross-over design. Following a 3-min break, the participants performed the target-tracking, a 10-min procedural, and a second target-tracking task. The displays were then switched, and the sequence was repeated. The order of display use was randomized and stratified by age, with participants assigned alternately according to a descending age order. A usability questionnaire was administered after completion.

The study was approved by the Institutional Ethics Review Board of the Institute of Science Tokyo (C2023-070). Written informed consent was obtained from all participants.

### Procedural task

The experiment was intentionally performed under non-optimal viewing conditions for PPD. The viewing position was set 100 cm horizontally from the display, with the line of sight directed 20° downward toward the screen center (Fig. 1C). The Stapes Challenge of the OtoSkills Trainer (Grace Medical) was used (Fig. 1D, sMovie 1). The participants inserted the long arm of a J-shaped metal hook into a base hole and engaged the short arm onto a horizontal protrusion using alligator forceps. Six hole-protrusion pairs were available. Once all completed, the hooks were removed and the procedure was repeated. The 10-min session was video-recorded, and the number of successfully completed tasks was determined from the recordings.

### Target-tracking task

To assess visual fatigue, a target-tracking task was performed before and after the procedural task (Fig. 1E, sMovie 2). Eye movements were recorded using a glasses-type eye tracker (NEON, Pupil Labs). The paradigm was adapted from a previously reported method [13]. Participants tracked alternating visual targets presented on a 27-inch display at a viewing distance of 60 cm while keeping their heads stationary. A total of 64 target pairs were presented per session.

Task-related saccades were extracted based on stimulus timestamps. Saccades with amplitudes of 5°–25° and durations > 100 ms were analyzed. The saccadic main sequence (amplitude vs. peak velocity) was calculated using robust linear regression (Theil–Sen method) with the intercept fixed at zero. The post-task-to-pre-task slope ratio was used as an objective index of oculomotor fatigue.

### Questionnaire

After performing the tasks using both displays, a questionnaire was administered to the participants. The questionnaire was specifically developed for this study. They were asked to rate each display on the following seven items on a scale of 5 (good) to 1 (poor): image resolution, 3D visibility, screen flickering, ease of procedure, concentration level, fatigue, and 3D motion sickness.

### Statistical analysis

Statistical analysis was conducted using GraphPad Prism 10 (GraphPad Software) or R software. Wilcoxon matched-pairs signed rank tests were used. *q* values were calculated using the Benjamini–Krieger–Yekutieli method. *P* or *q* values of less than 0.05 were considered statistically significant.

## Results

### Questionnaire

**Fig. 2:**
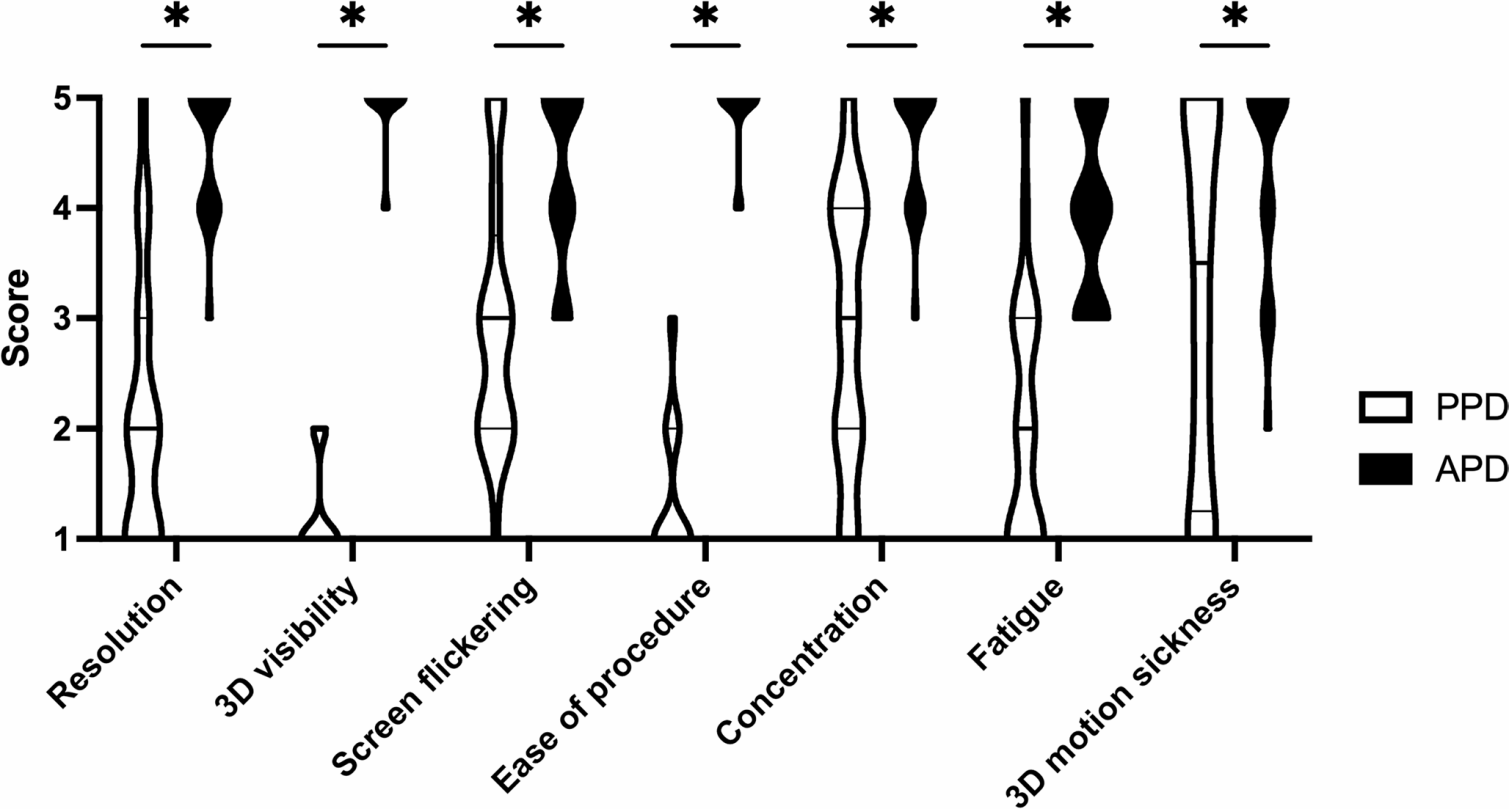
Questionnaire results. Participants rated each display on 7 items using a 5-point scale, ranging from 5 (good) to 1 (poor). The prototype active polarized 3D display (APD) outperformed the conventional passive-polarized 3D display (PPD) across all items (*n* = 24; multiple Wilcoxon matched-pairs signed rank test; *, *q* < 0.01)

The usability of the two displays is illustrated in Fig. 2. For PPD and APD, the median scores for image resolution, 3D visibility, screen flickering, ease of procedure, level of concentration, fatigue, and 3D motion sickness were 2 and 5 (q < 0.001), 1 and 5 (q < 0.001), 3 and 4.5 (q < 0.001), 1 and 5 (q < 0.001), 3 and 5 (q < 0.001), 2 and 4 (q < 0.001), and 3.5 and 5 (q = 0.004), respectively. Overall, APD outperformed PPD across all items, with particularly large differences in 3D visibility and ease of procedure.

### Procedural task completion count

**Fig. 3:**
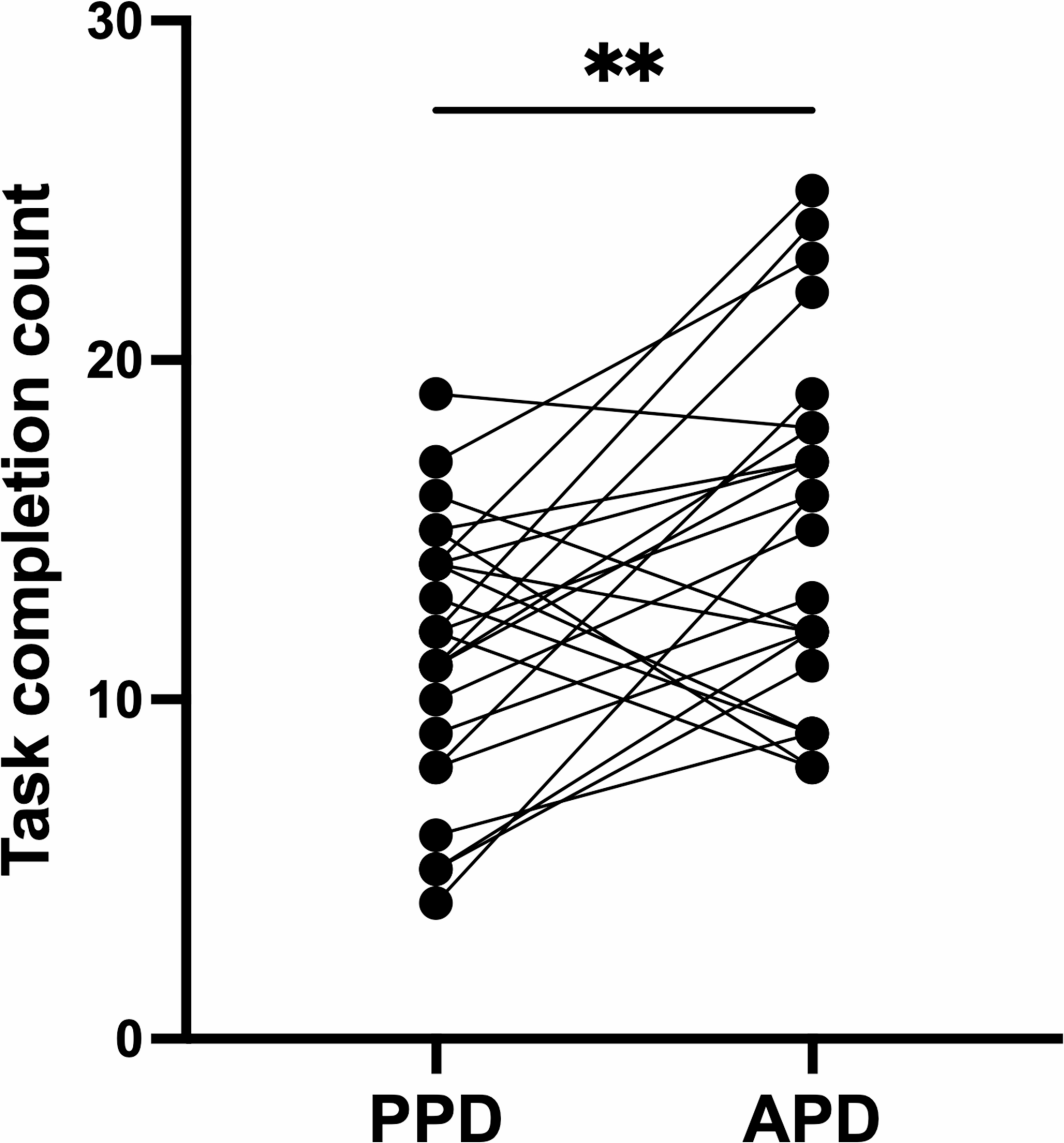
Procedural task success count. All procedural tasks were recorded, and the number of successful completions was counted. Across all participants, the prototype active-polarized 3D display (APD) resulted in a significantly higher number of successful task completions than the conventional passive-polarized 3D display (PPD) (*n* = 24; Wilcoxon matched-pairs signed-rank test; **, *p* = 0.008)

The number of successfully completed procedural tasks was determined by reviewing recorded video footage (Fig. 3). With the PPD, the median number of successfully completed tasks was 12 (range, 4–19), whereas with the APD it was 15.5 (range, 8–25). Task completion counts were significantly higher with the APD than with the PPD (*p* = 0.008).

### Target-tracking task

**Fig. 4:**
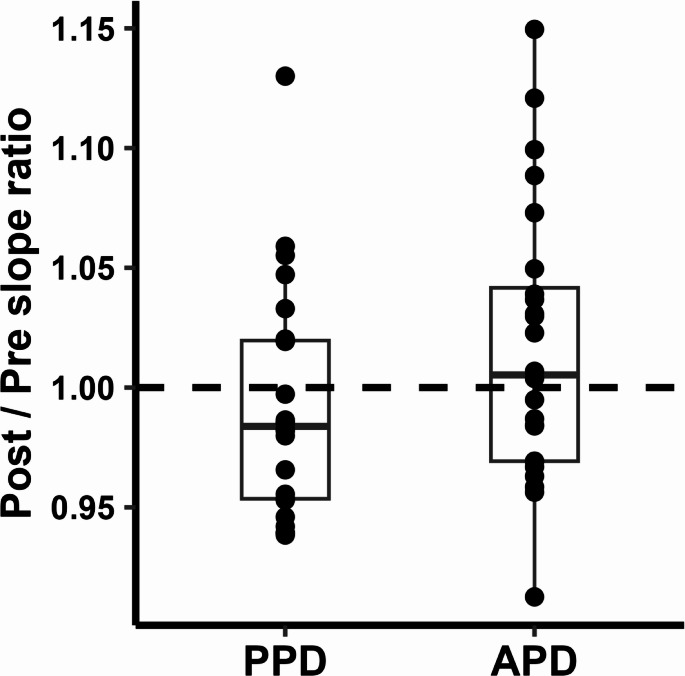
Oculomotor fatigue assessed by the post/pre slope ratio of the saccadic main sequence. Dot plots show individual post-task to pre-task slope ratios of the saccadic main sequence for the passive-polarized 3D display (PPD) and the active-polarized 3D display (APD). The box plots indicate the median and interquartile ranges for each condition. Values below 1.0 indicate a reduction in saccadic peak velocity after the procedural task, reflecting greater oculomotor fatigue. The dashed horizontal line denotes a ratio of 1.0. No statistically significant differences were observed between the PPD and APD groups (Wilcoxon matched-pairs signed-rank test, *p* = 0.188)

The post-task to pre-task slope ratio of the saccadic main sequence was used as an index of oculomotor fatigue, with values below 1.0 indicating a reduction in saccadic peak velocity after the task and thus greater fatigue (Fig. 4). The median ratios were 0.984 (range, 0.938–1.130) for the PPD and 1.005 (range, 0.913–1.150) for the APD. Although the ratio tended to be higher with the APD, indicating less fatigue-related reduction in oculomotor performance, the difference between the APD and PPD was not statistically significant (*p* = 0.188).

## Discussion

In this study, we used the ORBEYE exoscope system to compare the usability of two stereoscopic displays, a conventional PPD and an APD prototype, and demonstrated the APD’s overall superiority. The questionnaire results indicated that the prototype APD scored higher than the conventional PPD for all evaluated items. In particular, a marked difference was observed in the 3D visibility. In the experimental setup, the participants’ viewing position was set at 20° above the center of the screen. At this viewing angle, the conventional PPD fell outside the optimal vertical viewing range required for proper 3D perception. In contrast, the APD does not impose such limitations on 3D visibility in the vertical direction. A substantial difference was also noted in the ease of the procedure, which was likely related to the superior 3D visibility provided by the APD.

In an actual surgical setting, the display positioning is typically optimized at the surgeon’s eye level. However, assistants often share the same display, and their viewing positions are not necessarily optimal. Under such conditions, conventional PPDs might fail to provide adequate 3D visualization, whereas APDs could provide it even at non-optimal viewing positions. Therefore, the APD can accommodate a wide range of assistant viewing positions in the operating room.

In addition to neurosurgical and otologic procedures, the ORBEYE system is increasingly used in head and neck surgery [8–11]. In these procedures, surgeons often operate in a standing position, and eye height may change dynamically during the operation. Under such circumstances, APD is expected to offer superior usability for surgeons, owing to its flexibility in maintaining 3D visibility across varying viewing heights.

Objective performance measures supported the superiority of the APD prototype. The number of successfully completed procedural tasks was significantly higher with the APD than with the PPD in this laboratory-based simulated task. This difference is likely attributable to improved 3D visibility and greater ease of performing fine manipulations. Although these findings were obtained under experimental conditions, they may have clinical implications for otolaryngologic procedures requiring precise movements, such as middle ear surgery, delicate nerve dissection, or suturing, particularly when assistants operate from an elevated position during actual surgery.

In this study, a target-tracking task was performed before and after a procedural task to objectively evaluate ocular fatigue. Although the differences were not statistically significant, the slope of the saccadic main sequence was relatively preserved when the APD was used, whereas a slight decrease in the slope was observed when the PPD was used. Given that the questionnaire results indicated greater subjective fatigue with conventional PPD, this trend may reflect increased oculomotor fatigue associated with its use. However, the present results are not conclusive, and further investigation is required. In the current study, the procedural task was limited to 10 min for feasibility reasons, which may have reduced the sensitivity to detect clear differences in oculomotor fatigue between the display conditions.

Another method for viewing 3D images is head-mounted displays (HMDs). Using this approach, the images of the right and left eyes can be projected directly onto the corresponding eyes from the outset. Consequently, both surgeons and assistants can perceive 3D images regardless of their position, which is a key advantage. However, a previous study reported that although surgical performance does not differ between HMD and 3D displays, the use of HMD is associated with greater physical discomfort, facial discomfort, headaches, and ear discomfort [14]. In addition, head-mounted displays are expensive, making it difficult for all operating room personnel to wear them, and they also have the disadvantage of obscuring essential information, such as the patient’s vital signs. Therefore, 3D displays may be advantageous.

This study retains several limitations. First, the evaluation was performed at a single viewing position unfavorable for conventional PPD, and frontal viewing conditions were not assessed. Second, the small sample size and the brief, non-clinical procedural task might limit generalizability. In addition, a non-commercial APD prototype was evaluated, the performance of which potentially differing from that of commercially available systems. Finally, no washout period was included. Therefore, learning or adaptation effects during task repetition could not be excluded. Despite these limitations, this study provides preliminary evidence supporting the utility of APD in a 3D exoscope system and highlights its potential clinical applicability.

In conclusion, this study demonstrated that an active-polarized 3D display enhances the usability of a 3D exoscopic system. Such displays may be especially beneficial for individuals, such as standing surgeons and assistants, who must operate at varying eye levels. The application of active-polarization displays is expected to expand in future clinical settings.

## Electronic Supplementary Material

Below is the link to the electronic supplementary material.

Supplementary Material 1

Supplementary Material 2

Supplementary Material 3
