# Supplementary material for: Improved usability of an active-polarized 3D display in exoscopic surgery under non-optimal viewing conditions
Source: Eur Arch Otorhinolaryngol. 2026 Apr 23;283(8):5301–8. doi: 10.1007/s00405-026-10177-0 (PMC13407590; doi:10.1007/s00405-026-10177-0)
Supplement: Supplementary file 1 — Supplementary Material 1 [file 405_2026_10177_MOESM1_ESM.pdf]

Detailed Materials and Methods

Display settings, participants, and experimental procedures

We developed a 32-inch APD prototype. A commercially available 32-inch PPD (LMD-XH320MT, Sony, Japan) was used as the control. Both displays were connected in parallel to the ORBEYE 4K 3D exoscope system so that the same video output was displayed on each display. The comparison of two displays is shown below.

	**APD**	**PPD**
Size/Resolution delivered per eye	31.5”/3480×2160	31.5”/3480×1080
Brightness with 3D glasses	Adjust both to the same brightness	Adjust both to the same brightness
Refresh rate (panel)	≥120 Hz	60 Hz
Effective refresh rate (per eye)	≥60 Hz	60 Hz
Contrast Ratio	1000:1	1100:1
Latency	1 frame or less	1 frame or less

Participants were otorhinolaryngologists and head and neck surgeons. A total of 24 physicians participated in the study. This was an exploratory study, and no formal sample-size calculation was performed. The median participant age was 37 years (range: 26–59 years). The tasks performed by participants are shown in Fig. 1B. After receiving instructions for the procedural task, participants practiced it for 5 min without an exoscope before proceeding to the main experiment. After a 3-minute break, participants performed a target-tracking task, then completed a procedural task for 10 minutes, and then performed a second target-tracking task. The displays were then switched, and the same sequence of tasks was repeated. The order of display use was randomized, stratified by participant age. Finally, a questionnaire was administered to assess the usability of each display.

This study was approved by the Institutional Ethics Review Board of the Institute of Science, Tokyo (C2023-070). Written informed consent was obtained from all participants.

Procedural task

The experiment was intentionally performed under non-optimal viewing conditions for PPD. The procedural task was performed with the viewing position set 100 cm horizontally from the display and the line of sight directed 20° downward toward the center of the display (Fig. 1C). The Stapes Challenge of the OtoSkills Trainer (Grace Medical, Memphis, TN, USA) was used for this task (Fig. 1D). In this training kit, a hole is present at the base and a horizontal protrusion is positioned above it. Using alligator forceps, the participants inserted the long arm of a J-shaped metal hook into the hole and engaged the short arm on the protrusion. There were six pairs of holes and protrusions. The participants sequentially hooked a metal hook onto each pair, and once all six were completed, all hooks were removed, and the same procedure was repeated. During the 10-minute task period, the entire process was recorded on video, and the number of successfully completed tasks was counted by reviewing the recorded footage.

Target-tracking task

To assess the visual fatigue, a target-tracking task was performed before and after the procedural task (Fig. 1E). During the target-tracking task, the participants wore a glasses-type eye-tracking device (NEON, Pupil Labs, Germany) that recorded their eye movements. The target-tracking paradigm was adapted from a previously reported method for evaluating fatigue-related changes in saccadic dynamics [13].

The viewing position was set at a horizontal distance of 60 cm from the center of a 27-inch display. Participants were instructed to track the visual markers on the display using eye movements while keeping their heads stationary. The target presentation program was customized in Python. First, a white dot was displayed at the center of the display. Subsequently, a white cross (displayed for 850 ms) and a red dot (550 ms) were alternately presented. A white cross was presented randomly in one of the four corners of the screen. Following each white cross presentation, a red dot appeared 17.5° away from the preceding white cross in one of three possible directions relative to the white cross position: vertical, horizontal, or diagonal.

A total of 64 fixation-target marker pairs were presented in each session. The eye movement data exported from the NEON system included multiple event classifications, among which saccade data were used for further analysis. Each saccade record contained a timestamp, amplitude, duration, and peak angular velocity. For all the detected saccades, those whose timestamps corresponded to the presentation of the red target dot, as defined by the Python-based stimulus program, were selected as task-related saccades. Saccades with amplitudes between 5° and 25° were extracted, and saccades with durations of ≤100 ms were excluded. For each session, the saccade main sequence was plotted by relating saccade amplitude (°) to angular peak velocity (°/s). The slope of the main sequence was calculated using robust linear regression (Theil–Sen regression), with the intercept fixed at zero. The ratio of the post-task slope to the pre-task slope was calculated and used as an objective index of oculomotor fatigue.

Questionnaire

After performing the tasks using both displays, a questionnaire was administered to the participants. The questionnaire was specifically developed for this study. They were asked to rate each display on the following seven items on a scale of 5 (good) to 0 (poor): image resolution, 3D visibility, screen flickering, ease of procedure, concentration level, fatigue, and 3D motion sickness.

Statistical analysis

Statistical analysis was conducted using GraphPad Prism 10 (GraphPad Software) or R software. Wilcoxon matched-pairs signed rank tests were used. q values were calculated with Benjamini-Krieger-Yekutieli method. p or q values of less than 0.05 were considered were considered statistically significant.
